# The DDGP (cisplatin, dexamethasone, gemcitabine, and pegaspargase) regimen for treatment of extranodal natural killer (NK)/T-cell lymphoma, nasal type

**DOI:** 10.18632/oncotarget.11135

**Published:** 2016-08-09

**Authors:** Lei Zhang, Sucai Li, Sisi Jia, Feifei Nan, Zhaoming Li, Jingyu Cao, Shanshan Fan, Chao Zhang, Liping Su, Jinghua Wang, Hongwei Xue, Mingzhi Zhang

**Affiliations:** ^1^ Department of Oncology, The First Affiliated Hospital of Zhengzhou University, Lymphoma Diagnosis and Treatment Center of Henan Province, Zhengzhou, Henan, China; ^2^ Department of Hematology, Shanxi Cancer Hospital, Taiyuan, Shanxi, China; ^3^ Department of Oncology, Nanjing General Hospital of Nanjing Military Command, Nanjing, Jiangsu, China; ^4^ Department of Oncology, The Affiliated Hospital of Qingdao University, Qingdao, Shandong, China

**Keywords:** extranodal natural killer/T cell lymphoma, DDGP regimen, chemotherapy, gemcitabine, pegaspargase

## Abstract

Extranodal natural killer/T cell lymphoma (ENKL) is a high invasive disease with poor prognosis. Since there is no consensus on standard chemotherapy, we developed an original chemotherapeutic DDGP (cisplatin, dexamethasone, gemcitabine, and pegaspargase) regimen. We retrospectively analyzed 80 patients who received DDGP chemotherapy. The primary end point was progression-free survival (PFS) and secondary end points were overall survival (OS), complete response rate (CRR), and overall response rate (ORR). The one-year PFS and OS rates were 86.0% and 88.6%, and the 2-year PFS and OS rates were 81.40% and 87.1%, respectively. The ORR and CRR of DDGP chemotherapy were 91.3% and 60.0%. The major adverse events were myelosuppression, digestive tract toxicities, and coagulation disorder. No treatment-related deaths were observed. Our results suggest that the DDGP regimen is a high effective and safe treatment for ENKL.

## INTRODUCTION

Extranodal natural killer (NK)/T-cell lymphoma, nasal type (ENKL) is a rare subtype of non-Hodgkin lymphoma. It mainly occurs in the nasal/paranasal area, skin, lung, or gastrointestinal tract, and is much more common in Asia and Latin America than in Western countries [1–3]. ENKL accounts for 5% to 18% of all cases of non-Hodgkin lymphoma (NHL) [4–6]. ENKL is strongly associated with infection by Epstein-Barr virus (EBV). EBV-DNA viral load correlates well with clinical stage, response to therapy, and survival. The measurement of EBV viral load is important for diagnosis and disease monitoring [7–9].

The prognosis of ENKL is extremely poor under conventional anthracycline-based chemotherapy, such as CHOP (cyclophosphamide, doxorubicin, vincristine, and prednisone) and CHOP-like regiments. Most patients died within 2 years after diagnosis, with median survival of only 2-7.8 months under those regimens above [10–13]. This poor outcome is partly due to expression of P-glycoprotein by ENKL tumor cells, which results in multidrug resistance [14–15]. No chemotherapeutic regimen is considered standard for the treatment of ENKL, so oncologists endeavor to explore more effective chemotherapeutic regimens. In recent years, some L-asparaginase-based regimens have been attempted for ENKL, and promising responses were observed [16–20]. These regimens usually include those drugs which were generally unaffected by MDR pathways, such as methotrexate, L-asparaginase, dexamethasone, and so on. However, L-asparaginase has been associated with a high incidence of hypersensitivity reactions (73%), including life-threatening anaphylaxis. Additionally, L-asparaginase has a short half-life in vivo (20 hours), thus requiring daily administration [21]. These demerits restrict application of L-asparaginase.

As a pegylated form of L-asparaginase, pegaspargase has a longer *in vivo* half-life (357 hours), therefore requiring less frequent dosing, and has lower incidence of hypersensitivity reactions. Moreover, efficacy is similar to L-asparaginase at comparable cost [21]. The pyrimidine antimetabolite gemcitabine is demonstrated to be one of the most effective agents against malignant lymphoma when used either as monotherapy or as part of a combination regimen [22–24]. However, we are aware of no report examining the efficacy of chemotherapeutic regimens incorporating both gemcitabine and pegaspargase for the treatment of ENKL.

According to the half-maximal inhibitory concentrations (IC50s) of chemotherapeutic drugs on NK/T cell lymphoma lines, we developed DDGP (cisplatin, dexamethasone, gemcitabine, and pegaspargase) regimen, as a more effective and safer treatment for ENKL [25–26]. Previously, our center reported 100% ORR in 12 newly diagnosed stage II−IV ENKL patients treated with the DDGP regimen [27]. In addition, relapsed/refractory ENKL patients treated with the DDGP regimen exhibited ORR and CRR of 88.2% and 52.9%, with one-year OS rate and one-year PFS rate of 82.4% and 64.7% [28]. For further evaluation of DDGP efficacy and safety, we enrolled a larger cohort of ENKL patients (80) and conducted a retrospectively study assessing one- and two-year PFS and OS as well as ORR and CRR.

## RESULTS

### Patient characteristics

We enrolled 80 eligible patients from March 2010 to December 2014. Baseline patient characteristics are listed in Table 1. Median age was 43 years (range, 13 to 70 years) and the male to female ratio was 49:31. The primary involvement site was the upper aerodigestive tract in 72 patients and non-upper aerodigestive tract in 8 patients. B symptoms were observed in 38 patients (47.5%) and lactate dehydrogenase (LDH) was elevated in 35 patients (43.8%). EBV was positive in 77 patients (96.3%). Forty-eight patients (60%) were newly diagnosed ENKL, 9 were in first relapse, and 23 were in primary refractory state. Among the 30 patients who received chemotherapy or chemoradiotherapy as first-line therapy, 17 were treated with CHOP or CHOP-like regimen, three patients with the SMILE regimen (dexamethasone, methotrexate, ifosfamide, L-asparaginase, and etoposide), and seven with the VIPD regimen (etoposide, ifosfamide, cisplatin, and dexamethasone). The previous regimens were unknown in three patients. Among the 9 relapse cases, two were treated with radiation alone as the initial therapy.

**Table 1:** Clinical characteristics of patients with ENKL (n=80)

Characteristics	Values of n	Percent(%)
Sex		
Male	49	61.3
Famale	31	38.7
Age(years)		
Median	43	
Range	13-70	
Disease state		
Newly-diagnosed		
Stage I-II	20	25.0
Stage III-IV	28	35.0
Relapse	9	11.3
Refractory	23	28.8
Site(s) of invovement at diagnosis		
UNKTL	72	90.0
EUNKTL	8	10.0
LDH		
Normal	45	56.3
Elevated	35	43.8
B symptom		
Negative	42	52.5
Positive	38	47.5
IPI		
0	11	13.8
1	20	25.0
2	28	35.0
3	13	16.3
4	8	10.0
Prior treatment		
None	48	60.0
Radiotherapy alone	2	2.5
Concurrent chemoradiotherapy	12	15.0
Chemotherapy alone	18	22.5

Abbreviations: UNKTL Upper aerodigestive tract NK/T-cell lymphoma, EUNKTL Non-upper aerodigestive tract NK/T-cell lymphoma, IPI International prognostic index.

### Treatment responses and survival

The treatment responses are summarized in Table 2. The median number of DDGP regimen cycles per patient was 4 (range 3-6 cycles). The ORR was 91.3% (95%CI, 85.0% to 96.3%). Forty-eight patients (60.0%, 95%CI, 48.8% to 71.3%) reached complete response (CR) and 25 patients (31.3%) reached partial response (PR). Progressive disease (PD) was observed in four patients during therapy and stable disease (SD) in three patients.

**Table 2:** Summary of treatment outcomes

Response	All Patients (n=80)		Newly Diagnosed Stage I/II (n=20)		Newly Diagnosed Stage III/IV (n=28)		Relapse Or Refractory patients (n=32)	
	No	%	No	%	No	%	No	%
CR	48	60.0	14	70.0	17	60.7	17	53.1
PR	25	31.3	6	30.0	8	28.6	11	34.4
SD	3	3.8	0	0.0	0	0.0	3	9.4
PD	4	5.0	0	0.0	3	10.7	1	3.1
OR	73	91.3	20	100.0	25	89.3	28	87.5

Abbreviations:CR Complete response, PR Partial response, SD Stable disease, PD Progressive disease, OR Overall response.

**Figure 1:**
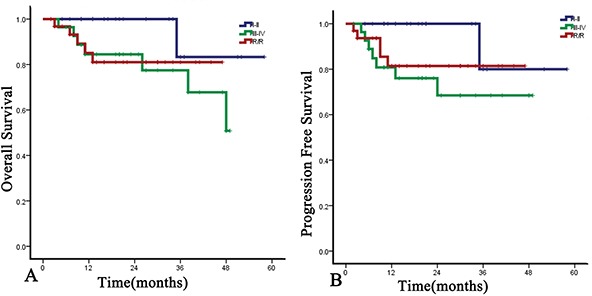
Kaplan-Meier estimates of overall survival (OS) and progression-free survival (PFS) of DDGP (cisplatin, dexamethasone, gemcitabine and pegaspargase) regimen for patients with newly diagnosed, relapsed and refractory ENKL **A.** Overall survival of DDGP on patients with newly diagnosed, relapsed or refractory ENKL. The 1-year and 2-year OS were 88.6% and 87.1%, respectively. **B.** Progression-free survival of DDGP on patients with newly diagnosed, relapsed or refractory ENKL. The 1-year and 2-year PFS were 86.1% and 81.4%, respectively.

**Figure 2:**
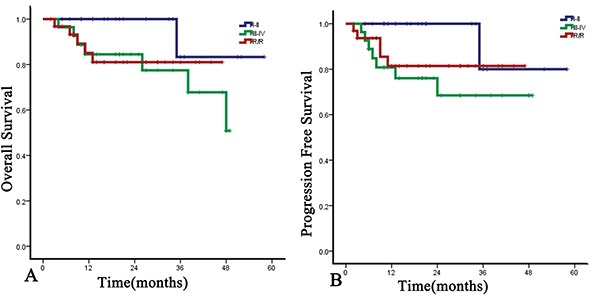
OS and PFS of DDGP chemotherapy on patients with newly diagnosed staged I-II, newly diagnosed staged III/IV, or relapsed/refractory ENKL, respectively **A.** OS of DDGP regimen on different disease status of ENKL. The 1-year and 2-year OS for patients with newly diagnosed stage I-II disease were 100% equally, and the 3-year OS was 83.3%. For the newly-diagnosed staged III/IV patients, the 1-year and 2-year OS rate were 84.5% equally. For the relapsed or refractory patients, the 1-year and 2-year OS were 85.1% and 81.0%, respectively. **B.** PFS of DDGP regimen on different disease status of ENKL. The 1-year and 2-year PFS for patients with newly diagnosed stage I-II disease were 100% equally, and the 3-year PFS was 80.0%. For the newly-diagnosed staged III/IV patients, the 1-year and 2-year PFS rate were 80.8% and 68.4%, respectively, and for the relapsed or refractory patients, the 1-year and 2-year PFS rate were 81.4%, equally. R/R, Relapse or refractory.

**Figure 3:**
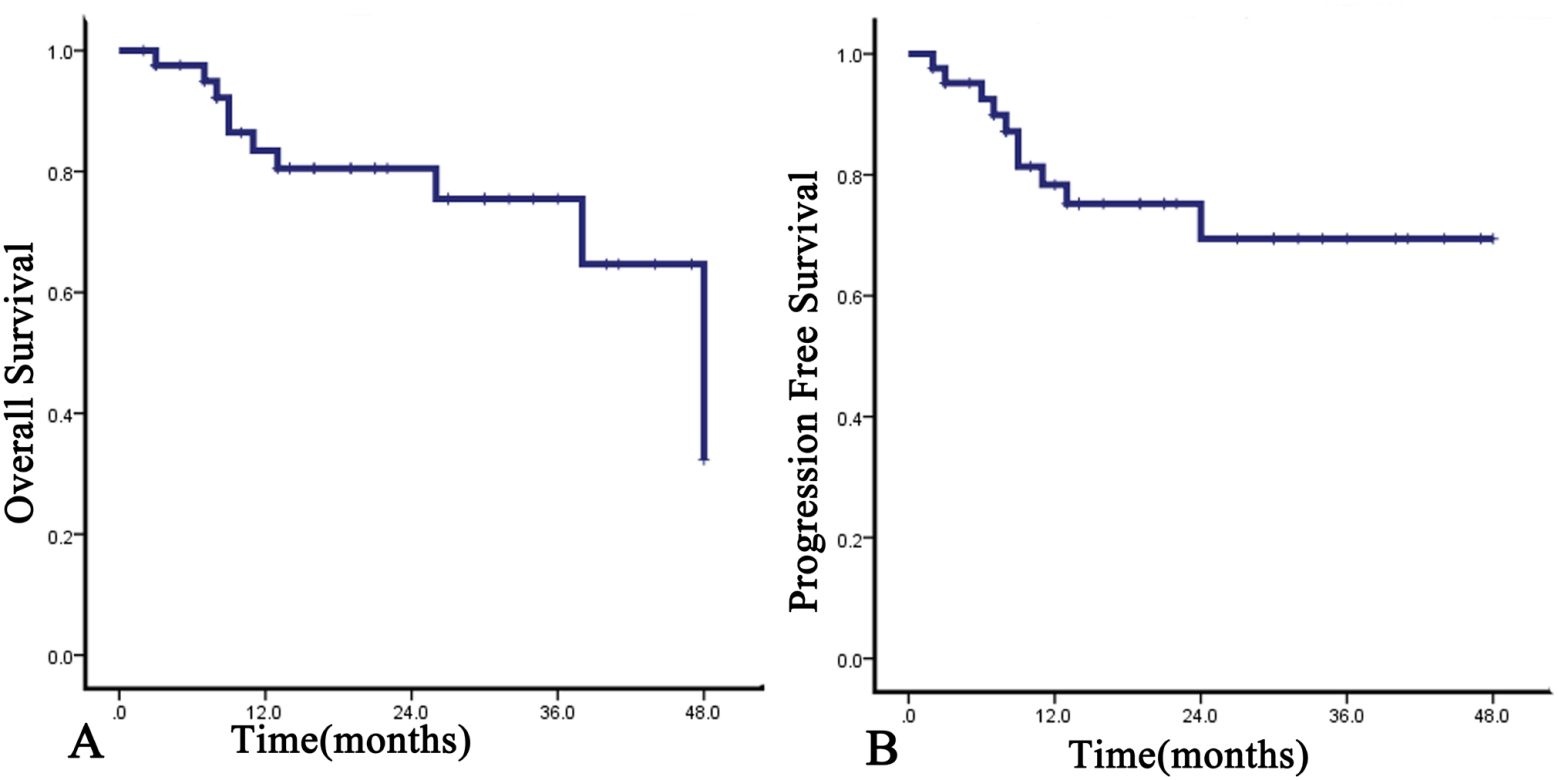
OS A. and PFS B. of DDGP chemotherapy on patients with newly diagnosed staged IV, relapsed or refractory ENKL The 1-year OS and PFS rates were 83.5% and 78.3%, respectively. The 2-year OS and PFS rates were 80.5% and 69.4%, respectively.

With the median follow-up time of 20 months (range, 2-58 months), the 1-year OS and PFS rates were 88.6% (95%CI, 84.8% to 92.4%) and 86.1% (95%CI, 82.0% to 90.2%), respectively, and the 2-year OS and PFS rates were 87.1% (95%CI, 83.4% to 91.4%) and 81.40% (95%CI, 76.3% to 86.5%) (Figure 1). For the newly diagnosed staged I/II patients, the ORR and CRR were 100% (20/20) and 70.0% (14/20), and the 1-year and 2-year OS and PFS rates were both 100%. The 3-year OS and PFS rate were 83.3% and 80.0%. The median OS and PFS points have not been reached. For the newly diagnosed stage III/IV patients, ORR and CRR were 89.3% (25/28) and 60.7% (17/28). The 1-year and 2-year OS rates were 84.5% equally, and the 1-year and 2-year PFS rates were 80.8% and 68.4%. For the relapsed or refractory patients, the ORR and CRR were 87.5% (28/32) and 53.1% (17/32). The 1-year and 2-year OS were 85.1% and 81.0%, respectively, and the 1-year and 2-year PFS rates were both 81.4% (Figure 2). For newly diagnosed staged IV, relapsed, or refractory patients (n=42), CRR was 54.8% (23/42), and 1-year OS and PFS rates were 83.5% and 78.3%, respectively (Figure 3).

We planned 3 cycles of the DDGP regimen before or after primary involved field radiotherapy for those patients with newly diagnosed stage I/II ENKL (n=20), but one patient did not return to hospital after 3 cycles. Finally, 19 newly diagnosed stage I/II patients received radiotherapy before or after the DDGP regimen. Four newly diagnosed stage III/IV patients received radiotherapy as salvage treatment after local PD, and two newly diagnosed stage III/IV patients received radiotherapy as consolidation therapy after remission.

Fifty-four patients are still alive, eight in relapse, 38 (38/80, 47.5%) with no evidence of disease (NOD), and eight (8/80, 10.0%) with PR. Ten patients (10/80, 12.5%) died from disease progression (DOD) and three from causes unrelated to disease (one from blood transfusion reaction at other hospital, one from gastrointestinal hemorrhage nine months after treatment completion, and one from adverse events of other salvage chemotherapy). Thirteen patients were lost to follow-up.

### Safety

Treatment-related adverse events are shown in Table 3. Grade 3/4 leukopenia and neutropenia occurred in 42 patients (42/80, 52.5%) and 52 patients (52/80, 65.0%), respectively. Grade 4 thrombocytopenia and anemia were observed in 28 patients (28/80, 34.6%) and four patients (4/80, 5.0%), respectively. With regard to digestive tract toxicities, 59 patients (59/80, 73.8%) experienced various degrees of nausea or vomiting and 24 patients (24/80, 30.0%) experienced grade 3/4 digestive tract toxicities. Two patients (2/80, 2.5 %) had grade 3 infection and 27 (27/80, 33.8%) had liver dysfunction with alanine aminotransferase (ALT) or aspartate aminotransferase (AST) elevation. No allergic reactions were observed, and no patient developed pancreatitis. Prolonged activated partial thromboplastin time (APTT) was observed in 39 patients (39/80, 48.8%), and hypofibrinogenemia was observed in 33 patients (33/80, 41.3%). One patient experienced cerebral hemorrhage with grade 4 thrombocytopenia, hypofibrinogenemia, and APTT prolongation. Hypoproteinemia was observed in 59 patients (59/80, 73.8%). No treatment-related deaths were observed.

**Table 3:** Toxicity profile of DDGP regimen

Toxicity	Toxicity incidence. NO.(%)				
	Grade 0	Grade 1	Grade 2	Grade 3	Grade 4
Hematologic toxicity					
Leukopenia	7(8.8%)	8(10.0%)	23(28.8%)	26(32.5%)	16(20.0%)
Neutropenia	9(11.3%)	5(6.3%)	14(17.5%)	31(38.8%)	21(26.3%)
Anemia	9(11.3%)	11(13.8%)	27(33.8%)	29(36.3%)	4(5.0%)
Thrombocytopenia	23(28.8%)	13(16.3%)	12(15.0%)	4(5.0%)	28(35.0%)
Digestive tract effect	21(26.3%)	20(25.0%)	15(18.8%)	22(28.5%)	2(2.5%)
Liver dysfunction	53(66.3%)	16(20.0%)	10(12.5%)	1(1.3%)	0
Increase in BUN	77(96.3%)	3(3.7%)	0	0	0
Infection	74(92.5%)	3(3.8%)	1(1.3%)	2(2.5%)	0
Hypoalbuminemia					59(73.8%)
Decrease in FIB					33(41.3%)
Prolonged APTT					39(48.8%)
Allergic reaction					0
Pancreatitis					0
Hemorrhage					1

Abbreviations: APTT activated partial thromboplastin time, FIB fibrinogen, BUN Blood urea nitrogen.

## DISCUSSION

DDGP regimen was developed according to the IC50s of chemotherapeutic drugs against ENKL tumor cells. Our previous reports showed that DDGP regimen was effective for ENKL [27–28]. This larger case series confirms these initial results, demonstrating one- and two-year PFS and OS rates >80% in a more heterogeneous cohort including newly diagnosed, relapsed, and refractory patients in all stages.

It is suggested that radiotherapy is effective in the early stage of localized ENKL [29–31], and radio-chemotherapy was reported to produce a greater CRR than chemotherapy alone [32]. Furthermore, patients who received ≥50 Gy radiotherapy tended to achieve superior local control rates compared to patients who received <50 Gy [33]. In view of these results, three cycles of DDGP chemotherapy were administered before or after radiotherapy as an integrated regimen for newly diagnosed staged I/II patients, with radiotherapy dose set at 50 Gy.

In the phase II trial of concurrent radiation and weekly cisplatin followed by VIPD chemotherapy in newly diagnosed, stage IE to IIE ENKL [34], the ORR was 83.3%. While there are no statistical differences between VIPD and DDGP regimens in ORR (25/30 vs. 20/20, P=0.067), CRR (24/30 vs. 14/20, P=0.315), 3-year OS rate (86.28% vs. 83.3%, P = 0.590), and 3-year PFS rate (85.19% vs. 80.0%, P = 0.401). It is noteworthy that there are several critical differences in patient characteristics between the two groups. First, among the 20 patients newly diagnosed with stage I/II ENKL in our trial, 75% were in stage II, compared to 50% in the VIPD study. Second, 20% of the patients were older than 60 years of age in our study, compared to 13% in the VIPD study. These factors could have influenced the outcomes of our study. In any case, longer-term and larger multicenter trials are warranted to directly compare outcomes for the VIPD and DDGP regimens.

In our study, the 1-year OS and PFS rates were 83.5% and 78.3%, respectively, for the newly diagnosed stage IV, relapsed, or refractory patients (n=42), obviously superior to the survival rate of the phase II Study of SMILE chemotherapy for newly diagnosed Stage IV, relapsed, or refractory patients by Yamaguchi et al [17] (83.5% vs. 55%, P=0.003 for OS; 78.3% vs. 53%, P=0.014 for PFS, by chi-square test). Alternatively, CRR did not differ between the phase II Study of SMILE and DDGP (44.7% vs. 54.8%, P = 0.370, by chi-square test). However, in the SMILE study, the severe infection rate was 61% (23/38), higher than in our study (2.5%) (23/38 vs. 2/81, P = 0.000 by fisher's exact test). Additionally, 1.5% of patients in the SMILE study developed grade 4 infection, a severe adverse event not seen in our DDGP trial. Similarly, the rate of grade 4 neutropenia of SMILE was higher than DDGP (35/38 vs. 22/81 in our trial, P =0.000 by chi-square test). Based on our results, the DDGP regimen has relatively lower toxicity and resulted in better long-term remission compared to the SMILE regimen.

The DDGP, VIPD, and SMILE regimens all have demonstrated better efficacy for ENKL than past treatments, but large-scale, prospective, and multiple arms trials are required to identify the optimal treatment for specific ENKL subgroups. Our center is leading a multicenter, randomized, controlled trial of the DDGP regimen as first-line therapy for newly diagnosed ENKL (Clinical Trials.gov ID, NCT01501136 and NCT01501149). This clinical trial was designed to compare the efficacy and safety of DDGP with VIPD, SMILE, and radiotherapy. The trail is nearing completion and the results will be reported soon.

In conclusion, the DDGP regimen is effective and safe for ENKL. Survival was superior to SMILE for newly diagnosed stage IV, relapsed, or refractory patients. Further, toxicities were tolerable and rates of severe adverse events lower than SMILE. The DDGP regimen is at least as effective as VIPD, warranting further comparative evaluations.

## MATERIALS AND METHODS

### Patients

Of the 80 patients included in this study, 20 were newly diagnosed in stage I/II, 28 newly diagnosed in stage III/IV, 9 had relapsed, and 23 were refractory to the first-line therapy. All received the DDGP regimen at some time between March 2010 and December 2014. Before treatment, hematoxylin-eosin (HE)-stained sections from all patients were histologically reviewed and confirmed by hematopathologists from our institution based on the WHO classification. Immunohistochemical staining was performed on formalin-fixed, paraffin-embedded sections with antibodies against CD3ε, CD2, CD56, CD20, CD43, perforin, and granzyme B. The presence of EBV was confirmed by in situ hybridization for EBV-encoded RNA (EBER) in the neoplastic cells on histopathologic examination. Written informed consent was obtained from all participants.

The pretreatment staging procedures included a review of medical history, physical examination, routine blood tests, and serum chemistry profile. Computed tomography (CT) scans of the head, neck, thorax, and abdomen were performed to determine the extent of the primary lesion. In addition, bone marrow aspiration and biopsy were carried out before treatment start. At the end of 2, 4, and 6 cycles and after treatment completion, CT scan and positron emission tomography (PET) were used if possible to detect residual mass and assess response to treatment.

### Study design and treatment

The DDGP regimen was administrated as follows: dexamethasone, 15 mg/m^2^ intravenously on days 1-5; cisplatin, 20 mg/m^2^ intravenously on days 1-4; gemcitabine, 800 mg/m^2^ intravenously on days 1 and 8; pegaspargase, 2,500 IU/m^2^ intramuscularly on day 1. Patients (n=80) received 3-6 cycles of the DDGP regimen. For patients newly diagnosed in stage I/II, 3 cycles of the DDGP regimen were administrated before or after primary involved field radiotherapy. The DDGP protocol was repeated every 3 weeks, and the treatment response was assessed every 2 cycles or at the end of treatment completion. As pegaspargase was in the DDGP regimen, every patient was informed to avoid stodgy foods during treatment to prevent pancreatitis. For patients who experienced grade 4 adverse events on a previous cycle, doses were reduced not more than 20% for the next cycle. Granulocyte colony-stimulating factor (G-CSF), interleukin 11 (IL-11), or recombinant human thrombopoietin (TPO) was given to patients who suffered neutropenia or thrombocytopenia as support therapy.

### Treatment response, staging and toxicity criteria

The revised Cheson's standard response criteria for malignant lymphoma was adopted to assess treatment response. CR was defined as disappearance of all evidence of disease and disease-related symptoms. PR was defined as ≥50% decrease in sum of the product of the diameters (SPDs) of masses and no new site. PD was defined as appearance of new lesions or ≥50% increase in the SPD of existing lesions. SD was defined as failure to attain CR or PR but not meeting the criteria for PD.

The new prognostic staging system (NSS) [35] for ENKL was employed in our study. Stages are defined as follows: stage I, lesions confined within nasal cavity or nasopharynx without local invasiveness (paranasal sinuses or bony or skin invasion); stage II, localized disease with local invasiveness (paranasal sinuses or bony or skin invasion); stage III, localized disease with regional lymph node involvement, such as cervical lymph node; stage IV, disseminated disease (lymph node on both sides of diaphragm, multiple extranodal site).

The toxicity of the DDPG regimen was assessed during every cycle from the first day of the regimen to one month after the last treatment. Adverse reactions were monitored by routine physical examination, routine blood tests, biochemistry, coagulation function test, urinalysis, and electrocardiogram (ECG). Toxicities were graded according to the National Cancer Institute Common Terminology Criteria for Adverse Events, Version 4.0. If an anaphylactic reaction to pegaspargase occurred, we stopped DDGP and turned to other treatment regimens.

### Statistical analysis

The primary end point was PFS, while secondary end points were OS, CRR and ORR at the end of treatment. PFS was measured from the date of treatment onset until disease progression or death from any cause. OS was defined as the time from the date of treatment onset until death or the last follow-up. ORR was defined as the percentage of patients who achieved CR and PR.

All data analyses were performed by using SPSS Statistics 21.0 software. Overall survival and PFS were estimated by the Kaplan-Meier method. Survival curves were compared between groups by log-rank test. The comparison of rates for different regimens was performed by chi-square test. For all tests, a P value less than 0.05 was considered statistically significant.
